# Developing a Mentorship Program in Laos

**DOI:** 10.3389/fpubh.2017.00145

**Published:** 2017-06-30

**Authors:** Helen Nita Catton

**Affiliations:** ^1^Primary Health Care Program, Save the Children International, Luang Prabang, Laos

**Keywords:** mentorship, capacity building, maternal and newborn health providers, supportive supervision, coaching

## Abstract

Skills strengthening and capacity building for maternal and newborn health (MNH) providers are essential to ensure quality care for mothers and newborns. There is, however, limited research regarding what constitutes an effective model in low-income countries. The Lao People’s Democratic Republic (Laos) has some of the region’s worst outcomes for neonatal and maternal mortality. Moreover, with a 23-year hiatus in midwifery training, which ended approximately 7 years ago, there is a cadre of new and inexperienced midwives in practice without support systems, skills, or continuing professional development opportunities. Traditional didactic teaching methodologies prevail in Laos, but with little evidence of efficacy. As an alternative model, Save the Children International has been implementing a mentorship approach for MNH providers in two provinces in northern Laos since January 2016, with technical guidance and funding from the United States Agency for International Development-supported global Maternal Child Survival Program. This community case study will describe and reflect on the approach by highlighting the need and rationale for mentorship, followed by a description of the program’s core components and the results observed so far. Lessons learned and the application of the approach to different contexts and health-care professionals, considering both constraints and opportunities, will be discussed.

## Introduction

Laos is a landlocked country in Southeast Asia. The population of 6.8 million is made up of over 49 different ethnic groups. Seventy-one per cent of the population live in rural and remote areas with limited access to health services. Only approximately 41% of women give birth in a health facility (1). This is one of the lowest rates globally as cited in the United Nations Children’s Fund database. According to national statistics, the current neonatal mortality rate is 32 per 1,000 live births and the maternal mortality rate is 357 per 100,000 live births (1). However, it is estimated that rates are much higher in rural areas where the majority of the population resides.

The Save the Children Primary Health Care program has been implemented in Laos since 1992. The program has achieved results by investing in strengthening the building blocks of an integrated health system, focusing on capacity building of district health managers, health staff, and community volunteers (2). Strengthening of maternal and child health services has been an integral part of the Primary Health Care program since inception. In 2016, there was an opportunity to further this work with support from the United States Agency for International Development (USAID) Maternal Child Survival Program (MCSP). At the time, the Ministry of Health (MoH) was planning to develop supportive supervision for midwives, and it was hoped that learning from the mentorship approach could inform this model.

The MCSP is a global USAID initiative across 25 countries to reduce maternal, child, and neonatal deaths. In Laos, the MCSP implementation began in January 2016 as part of the Save the Children Primary Health Care program. The focus is on building skills for mentorship within the health system in order to develop the capacity of maternal and newborn health (MNH) providers. The international partnership affords technical expertise from a consortium of partners, including Jhpiego, an international non-profit organization affiliated to the John Hopkins University focused on women’s health and well known for expertise in training.

The term “mentor” derives from Homer’s Odyssey and differs from teacher–student relationships by way of the mentor being connected with a mentee in “both cognitive and affective domains” (3, 4). Mentorship is a feasible model of continuing professional development and supportive supervision for MNH providers. Mentorship in Laos involves participatory learning on the job and a reciprocal relationship between the mentor and the mentee encompassing coaching, demonstrating, providing constructive feedback, and planning for action. The approach in Laos is unlike formal mentorship whereby a senior mentor is selected by a junior colleague with a specific time-limited learning agenda. Instead, the approach focuses on peer to peer coaching in practice on a continuing basis as a model for supportive supervision. The evolution and development of the program thus far is described below and includes the following key activities in two distinct stages:

Stage one—developing Laos mentors and implementing mentoring visits to district facilities
Stage two—upgrading skills of select mentors to become trainer mentors through Training of Trainers and creating facility-based district mentors to ensure mentoring becomes part of sustainable daily practice, institutionalized in the district facility.

## Nature of the Problem and Rationale for Developing a Mentorship Approach

For 23 years, there was no midwifery training in Laos. In 2008, there were only 100 midwives in the country. In response, the government created the *Skilled Birth Attendant (SBA) Development Plan (2008–2011)* (5), with the aim to develop 1,500 new midwives by 2015. As a 2014 review of the development plan noted, the rapid, didactic training produced a cadre of young, inexperienced, and unskilled midwives deployed to remote districts and health centers without support or supervision, unable to provide quality care at the time of birth (6). A similar challenge was reported in Ethiopia, where rapid training produced midwives lacking competencies to deliver quality care (7). In Laos, the terms SBA and midwife are used interchangeably and refer to nurses who have completed a 1-year midwifery course as well as non-professionals completing a 2-year direct entry course. For the purposes of this article, the sole term “midwife” will be used.

The Laos government recognized the need to improve maternal and newborn care by developing capacity among MNH providers and developed two key strategies:

1. The Lao PDR MoH (2016) *National Reproductive, Maternal, Newborn and Child Health (RMNCH) Strategy and Action Plan 2016–2025* (8),
2. The Lao PDR MoH (2016) *Midwifery Improvement Plan 2016–2020* (9)

Save the Children International aims to support the MoH in achieving the objectives laid out in these key strategies by developing, implementing, and testing a capacity-building mentorship approach for MNH providers. There is a critical need to develop an effective model of capacity building and competency development for MNH providers in all levels of the system. A Cochrane review (2015) suggests in-service training improves outcomes for newborns but additional studies are required (10).

Traditional didactic models of pedagogy prevail in Laos but there is little evidence of their effectiveness (7, 11, 12). Didactic training focused on cognitive domains may not effectively prepare providers for the multidimensional complexity of clinical practice (13). A mentorship approach was developed that uses participatory learning through on-site coaching combining skill strengthening and support as a model of continuing professional development and supportive supervision within the facility, with the aim to improve the quality of care at the time of birth. Although mentorship has been implemented in other contexts, the Laos program focuses on full integration of maternal and newborn care, building skills and competencies through on-site coaching, and the removal of hierarchies to facilitate peer to peer learning which is new for this context. Furthermore, building the training capacity within the facility among a cadre of district mentors and ensuring continuing professional development and daily supportive supervision is a change from the traditional model of external trainers providing short courses.

## Methods

### Stage One

#### Developing Mentors and Tools for Mentorship

An initial workshop was held in February 2016 to work with 15 government-selected MNH providers (11 provincial and 4 district staff) to design the mentoring approach and build capacity to become mentors. The 15 government employees comprised 3 pediatricians, 2 nurses, 1 midwifery teacher, 2 obstetrics/gynecology physicians, and 7 midwives. The workshop was led in a participatory way to ensure that the mentors were fully engaged in developing the program and that it was appropriate to context, feasible, and acceptable. Empowering the mentors to shape the approach ensured their commitment and motivation. Despite many of the mentors being experienced educators in traditional didactic teaching methods, they very quickly took on board the participatory approach.

Initially the mentors’ clinical skills for a normal delivery and when the baby is not breathing were standardized using the guideline for practice (outlined further below). From this foundation, mentoring skills were developed, including effective facilitation skills, coaching, demonstrating, providing feedback, and action planning. These skills were then field tested by the mentors and improved and finalized during the workshop. To support the implementation of the mentorship approach, a number of tools were created including two integrated in-service guidelines for normal delivery and when the baby is not breathing. The first draft of the guideline was based on global standard guidelines and fully aligned with the MoH/World Health Organization Essential Early Newborn Care (EENC) guidelines and policy (14). Table 1 (below) highlights the nine key standards of the guideline for normal delivery when the baby is not breathing.

**Table 1:** Nine key standards of the integrated in-service guideline for normal delivery when the baby is not breathing.

1.	Monitor woman in labor using the partograph
2.	Preparation just before birth
3.	Assisting birth
4.	Immediate essential newborn care
5.	Newborn resuscitation—Airway and stimulation
6.	Newborn resuscitation—Bag and mask ventilation
7.	Active management of third stage of labor
8.	Post-delivery tasks
9	Infection control and routine procedure after birth

The draft guideline was shared with mentors for their input from practical experience. It was important to be realistic and understand contextual constraints. During the workshop, the guideline was reviewed and updated with input from the mentors. This process continued until a consensus was achieved and a practical, feasible clinical guideline created. A facility action plan tool was developed in line with global quality improvement tools which includes three domains; capability (knowledge and skills), opportunity (enabling environment), and motivation. The tool is used by staff to develop facility quality improvement action plans. The benefits of a combined mentoring and quality improvement approach have been demonstrated in similar programs in Rwanda (15). Due to the low number of facility deliveries and the need to develop skills, simulation using anatomical models Mamanatalie and Neonatalie are used for practice. These are simple and effective models that mentors and mentees learned to use quickly and successfully. Mentoring includes all four domains of simulation fidelity, namely, case study, role-play, focused task/skill practice, and full simulation (13).

#### Clinical Approach: Integrated and Comprehensive

An essential element of the program is the full integration of maternal and newborn care. To date, these have been separated under education, policy, and practice. For example, in Laos, the teaching of emergency obstetric care and EENC is disconnected and maternal and newborn care objectives are distinct under the new RMNCH Strategy (2016–2025) (8). In practice, a midwife may call a pediatrician to assist with newborn resuscitation if she is not confident, resulting in delays in life-saving care. Where mentorship differs is in the inclusion of both mother and newborn in the approach to care. The clinical guideline and standard of practice used in mentoring integrates maternal and newborn components equally, starting with the partograph (the vital sign tool for mother and newborn through labor and birth) and labor monitoring. The full integration of maternal and newborn care is an innovative and important development (16). The comprehensive clinical approach includes the following key components: respectful maternal care, ensuring good communication with the mother (an important part of patient perceived quality of care) (17), labor monitoring using the partograph, preparation before birth, safe delivery, early essential newborn care/newborn resuscitation, active management of the third stage of labor, skin to skin, early breast feeding, infection control, and correct disposal of waste.

#### Mentoring Visits to District Facilities

Following the workshop from March to July 2016, the 15 mentors provided mentoring visits to 10 district hospitals in two provinces to support skills strengthening as a model of collaborative education. Two mentors and one or two Save the Children staff spent 2 days in each facility. Mentoring activities were held in the delivery room with mothers during labor and delivery when possible, as evidence suggests the benefit of a real environment for practice (12, 18). Activities also involved demonstration and role-play practice in small groups (1 mentor:3 mentees) with coaching and feedback. The focus was on two clinical scenarios: normal delivery with baby breathing and normal delivery with baby not breathing. Strengthening foundational skills was the priority to ensure safe delivery and preventing complications of birth. Following clinical skill practice, a group feedback session and action plan to address issues and skills gaps was developed. Each facility was visited once every 2–3 months in a low–dose, high-frequency approach which is more effective than annual trainings (12, 19). The availability of mentors to undertake visits due to other commitments and responsibilities in their own facility was a challenge and catalyzed a review of how to successfully develop the mentoring approach going forward.

### Stage Two

#### Institutionalizing Mentorship in the Facility and Building District Level Capacity

**Figure 1:**
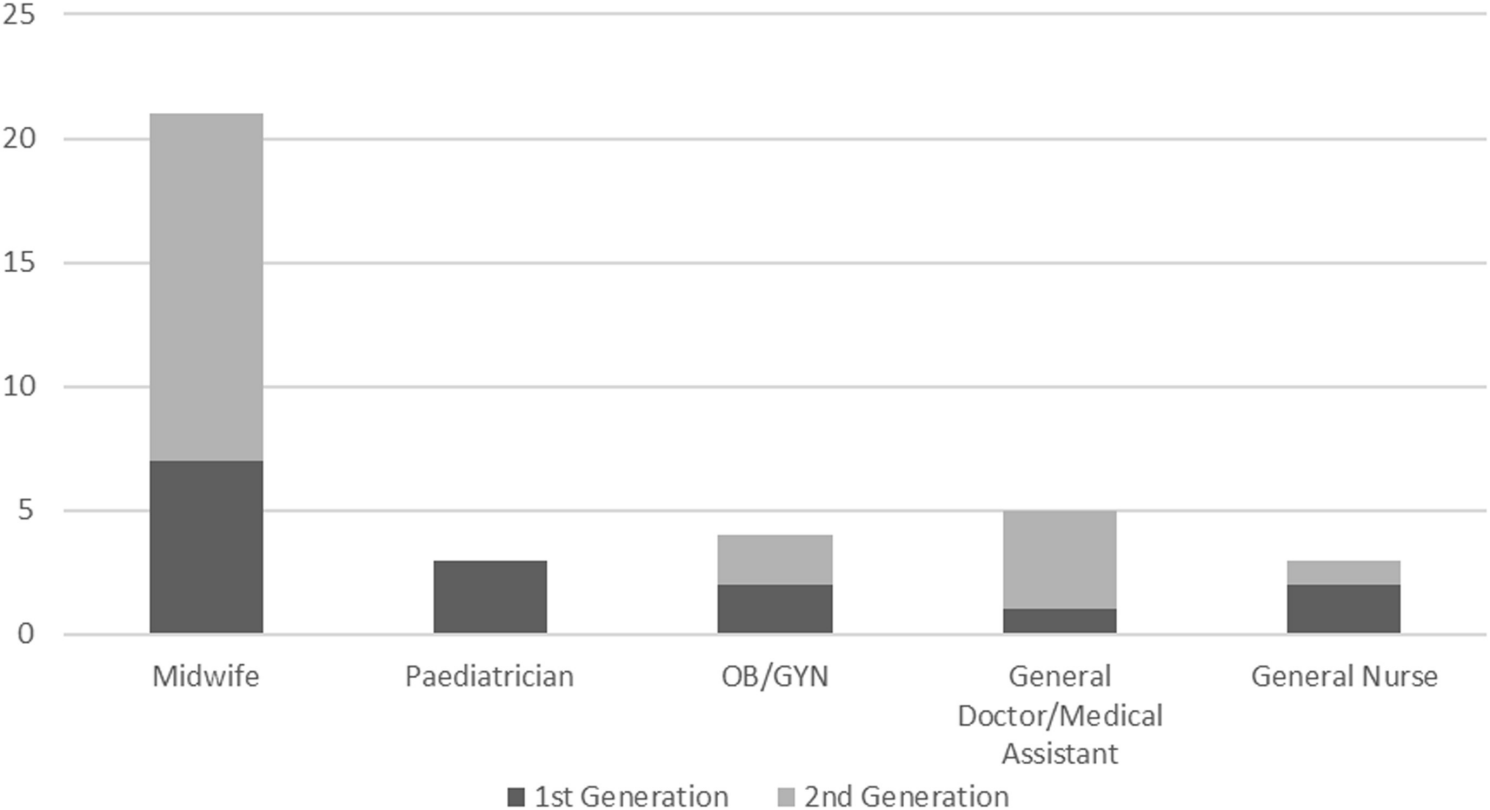
Distribution of the professional roles of the first- and second-generation mentors.

While quarterly mentoring visits with the original mentors (predominantly from the province) were well received, challenges of time and availability were noted. Thus, the opportunity was identified to fully institutionalize mentoring and build capacity in the district facility by developing district level mentors. Mentors within the facility further augment the low–dose, high-frequency approach by providing more opportunities for specific skills to be practiced frequently. District mentors also ensure on-site continuous support for mentees and quarterly practice drills for rare events, such as newborn resuscitation and management of postpartum hemorrhage. Criteria for selection and the roles and responsibilities of district mentors were developed in partnership with district health managers and district hospital directors. Two staff from each facility were selected by district managers to be the new district level mentors. The priority was on midwives and MNH providers active in the delivery room who most often facilitate deliveries. Figure 1 below shows the graph and distribution of mentors’ profession from the first generation (February 2016 cohort) and the second generation of predominantly district level staff (August 2016 cohort).

#### Training of Trainer Mentors and Developing District Mentors

To ensure sustainability and Laos ownership, it was necessary to develop Laos trainers of new mentors. A specific training of trainers was convened for 5 days and included 8 of the original 15 mentors who showed particular strengths in their leadership and mentoring of others. The eight trainers include four midwives, one pediatrician, one obstetrics/gynecology physician, and one nurse. The 5-day trainers’ workshop included standardizing the trainers’ clinical skills, effective facilitation skills, and microteaching practice, which involved developing lessons plans and peer-teaching with feedback sessions and daily de-brief meetings. The trainers built competencies to develop a new generation of mentors. Focus was on interactive participatory learning consistent with the mentorship approach itself. Following the trainers’ workshop, a second 5-day workshop was held to train the nominated district MNH providers to become new mentors. The workshop focused on clinical skills building and the mentoring skills of coaching, demonstrating, feedback, and action planning. The seven building blocks (skills) of mentoring are outlined in Table 2. From these seven building blocks, standards have been developed to measure progress in mentoring skills. Full competence in all seven standards will be developed with experience over time.

**Table 2:** Seven building blocks and standards of mentoring.

**Create a good learning environment**		
Coaching	Demonstrating	Feedback and assessment
Action planning	Reflect on own progress as a mentor	Safety of mother and newborn

The duration of both workshops of only 5 days was a limitation. It is acknowledged the process of becoming a confident and competent mentor takes time, as Benner describes in the transition from novice to expert (20). While the workshop established foundational skills, further support and development of mentoring skills will be required. A majority of district level mentors had limited prior experience as teachers or educators and are, therefore, starting a new role. This differs from the original 15 mentors who were predominantly experienced provincial educators, regularly engaged in teaching or supervising others in their hospital and in the training school.

#### Continuous Mentoring in the District Facility

With two district mentors in every facility, mentoring and coaching is part of daily activities with mentors supporting their colleagues to build skills, using opportunities during real deliveries. District mentoring allows 1–1 or 1–2 coaching in practice rather than small group role-playing as before. Identification of skills gaps among mentees is based on the clinical guideline as the standard tool for practice. District level mentors ensure the institutionalization of mentoring and sustainability in the facility. Challenges will be garnering consistent support for the mentor role from the district hospital director, the district health manager, and staff.

#### Buddy System of Support and Capacity Building

A key element of the mentorship approach is ensuring systems and networks of support and continuous professional development. This is important not only for mentees but also for mentors and trainer mentors. Supervision can improve performance and facilitate retention of staff (21) but supervisors also require support to progress (22, 23). To address this need, a buddy system of support was developed whereby the trainer mentor is assigned as a buddy mentor for two to four new mentors in the districts. The pairing was intentional to ensure continuity from the workshop through to ongoing buddy support.

The role of the buddy mentor includes making on-site visits to the district every quarter to assess progress and support the new district mentors in their roles. These visits provide the opportunity for the trainer mentors to support focused mentoring skills practice with the new mentors and to guide skills drills for rare events, for example newborn resuscitation. The importance of skills drills to build competencies in obstetric emergencies has been demonstrated (18). Finally, problems and challenges encountered by the new mentors are shared and solutions found collaboratively. Between on-site visits phone-calls are made to discuss progress or specific challenges. A quarterly mentor review meeting is held at the province which includes mentors and provincial and district leadership. The purpose of the meeting is to provide an opportunity for mentors to share feedback and lessons learned and to solve problems and challenges together with the district hospital directors. Results are presented and districts develop their facility action plans ensuring data are used for action.

Supervision in the form of on-site support can improve quality of care by improving the knowledge and skills of providers (24, 25). The system of support developed in the mentoring program can inform models of national supportive supervision, which have yet to be fully developed in Laos. In the current system, there are quarterly supervision and monitoring visits from the province to the districts, and the districts to the health centers. However, the focus tends to be observation and surveillance rather than support and capacity building, as has been noted elsewhere (25). Thus, there is an opportunity to incorporate a mentoring approach to these routine visits. The benefit of this approach has been documented in the mentoring and enhanced supervision of health centers program in Rwanda (15).

The two stages of the program, namely, developing the initial mentors and tools for implementation, followed by institutionalizing mentoring in the district facilities with a buddy system of support have been outlined above. As the approach is new for Laos, the importance of learning from implementation is the key. Together with key stakeholders and the MoH, the approach will be refined to develop a minimal package that can be replicated within the country context. Table 3 summarizes the key elements of the program.

**Table 3:** Key program elements of mentorship.

Essential components	Laos mentorship examples
Pedagogical style	Participatory learning in practice
Method of implementation	During real delivery or simulation using anatomical model (Mamanatalie/Neonatalie)
Location	In the delivery room
Frequency	Low-dose high-frequency (Specific skills practised frequently)
Development and retention of skills	Skills drills for rare events, e.g., newborn resuscitation and continuous mentoring skills development with buddy support
Institutionalized and sustainable	District level mentors in the facility ensuring mentorship are part of everyday practice
	Laos trainer mentors equipped to train new mentors
Support	Buddy system of trainer mentor supporting district mentor. District mentor supporting facility staff (mentees). Model of supportive supervision
Integrated	Maternal and newborn together
Comprehensive	Includes respectful maternal care, labor monitoring using partograph, active management of the third stage of labor, skin to skin, early breast feeding and infection control, and disposal of waste
Reflective, adaptive, and reproducible	Process documentation, regular mentor feedback, and review meetings to share lessons learned, inform continuous revisions of the approach, develop concise model adapted to context for reproducibility/scalability

## Results

**Figure 2:**
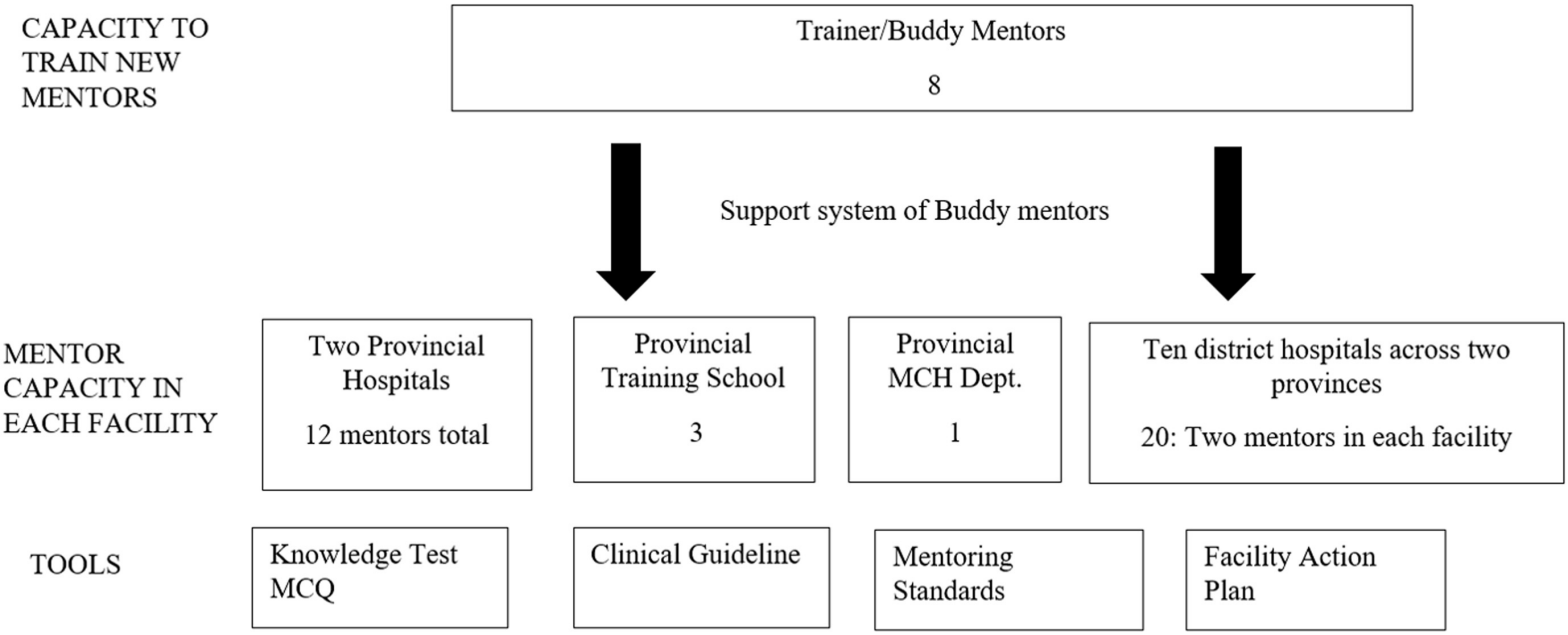
Diagram of the Mentor program showing mentor capacity in the facilities and tools for implementation.

In Laos, the mentorship program has developed eight Laos trainer mentors through a training of trainers’ workshop. The new trainer mentors have in turn developed 21 new mentors ensuring 2 mentors in every district facility. In total, the program has 36 mentors located in 14 facilities: two provincial hospitals, the training school, a provincial maternal child health center, and 10 district hospitals. Tools have been developed to support implementation of the approach including the clinical guideline, mentoring standards, and action planning tool. Development of the buddy system ensures support for trainer mentors, district mentors, and mentees. Figure 2 below shows a diagram of the mentoring program.

A limitation was the delay in establishing the monitoring and evaluation framework, which resulted in an absence of an initial baseline from the first cohort of mentors. However, qualitative evidence of improvements has been noted through data, case-studies, and service delivery readiness. Indicators on service provision from exit interviews with mothers and patient chart reviews, including the partograph and service delivery readiness, are being tracked quarterly. Results obtained from the second mentor workshop demonstrated improvements in pre and post-test knowledge among mentors from 55 to 83%. Clinical skill attainment measured in an objective structured clinical exam verified that the new mentors had obtained at least 67% proficiency in normal delivery when the baby is not breathing. Our aim is for 80% proficiency, and we will work to ensure all new mentors reach this standard by the end of the year. Initial results of the mentees’ clinical exam showed 14% were able to perform the steps of a normal delivery with the baby not breathing at a pass level of 80%. Three months later this figure has improved to 17%. Further incremental progress and skills development of mentees is predicted but will take time. An additional unanticipated challenge has been tracking a consistent cohort of mentees over time due to staff having numerous responsibilities both within the facility and externally. As a consequence, approximately 30% of the mentee cohort are taking the clinical exam for the first time each quarter, which results in the rate of progress being slower than would otherwise be expected. Positively, it means that more MNH providers have capacity-building opportunities and are engaged in the mentorship program. Encouragingly, mentoring skills of the mentors have shown greater progress. At baseline, 7% of new mentors gained a pass of 80% or more on their mentoring skills. However, with on-site coaching from the buddy mentor, 3 months later 72% were able to achieve 80% proficiency in their mentoring skills.

The institutionalization of mentoring in the facility with district mentors builds ownership and sustainability of the program. Furthermore, senior trainer mentors have independently taken mentorship to new districts as part of their capacity-building/training responsibilities under the provincial health department. At the central level, fifteen University of Health Sciences obstetrics/gynecology faculty underwent mentorship training to equip them to mentor residents using the approach and, in the longer term, build the residents’ skills in mentoring to mentor medical students in pre-service education. Integration of mentorship into the provincial health system through the routine monitoring and supervision visits has been initiated and will be measured through supervision visit reports and skills outcome measures. This will include outcome measures on reach when mentors independently apply mentorship in new geographical areas.

## Discussion

Initial results from implementation in Laos have generated interest among provincial and national leadership for mentorship as a feasible, effective, and acceptable model of skills strengthening and capacity building for MNH providers. The study group is small in order to pilot and develop the intervention before moving it out more widely. Documentation of lessons learned has informed adaptations and changes to the approach. Initial constraints included the lack of skills and experience in providing feedback and action planning among both mentors and facility staff. It was soon apparent that these skills are undeveloped in Laos. To overcome this, focused teaching was provided to mentors and mentees on feedback and action planning, which included problem identification, root cause analysis, and solution development. These sessions were subsequently included in all mentor workshops as core topics.

The availability of provincial level mentors to join mentoring visits due to work demands and other responsibilities was an initial constraint highlighted above. This has largely been overcome by the development of district level mentors. The abundance of programs and interventions both from the MoH and development partners results in a competitive programing climate. The simultaneous creation of new tools, guidelines, and checklists, rapidly developed and often later discarded, causes a guideline/checklist fatigue. This is often detrimental to effective implementation where programs overlap or staff are overwhelmed with numerous responsibilities.

The creation of district mentors was a key strategic decision which ensures institutionalization and sustainability of the mentorship approach in the facility. There are, however, new challenges to overcome including the acceptability among facility staff of peer learning where a tradition of being reliant on hierarchical systems and a provincial “expert” is the norm in Laos. This may be exacerbated by staff not being engaged or willing to change old practices or behavior. The mentorship program breaks the hierarchical mold but will require support from facility management for the mentors to perform their roles effectively. The long-term benefit of institutionalized facility-based mentoring, continuous professional development, and supportive supervision is significant and, therefore, perseverance with the model will be critical. Indeed, ineffective supervision has been found to be one factor influencing the lack of competent SBAs (26).

The loss of an initial baseline at the outset of the program due to delays in developing the monitoring and evaluation framework is a limitation. However, a baseline for 12 facilities has now been achieved. Furthermore, with multiple similar programs occurring concurrently, for example, coaching in EENC, it is difficult to verify whether improvements are a result of mentorship exclusively. There is a blending of effects that needs to be accounted for in any summary of results. Recognition that district mentors will need further support to develop their proficiency in mentoring skills and gain experience in this role is important. Similarly, improvements in the skills and capacity of mentees will also take time as discussed above. Furthermore, ensuring skills developed using the Mamanatalie anatomical model are transferred to real deliveries will need support and encouragement. Changing behavior is challenging and improvements may not be evident immediately. The practical implications include MNH providers with the skills and capacity to provide quality care at the time of birth for mothers and newborns and an effective model that bridges the gap of rapid didactic training of midwives which has left many without skills, confidence, or experience.

Mentorship can inform the current quarterly MoH supervision and monitoring system and guide it to a supportive and capacity-building approach, moving away from surveillance and inspection. This will be beneficial for the new MNH providers who are frequently required to work alone in health centers and are often deployed to remote contexts after graduating, without sufficient follow-up. Mentorship can be applied in other contexts and with other health-care providers. For example, mentorship has been shown to be effective with physicians in the care of childhood illnesses using the integrated management of childhood illnesses in Rwanda (27). However, it is important to shape the model to the particular context. From our experience, this is achieved most effectively by ensuring the mentors themselves are partners in developing and modifying the approach relevant to local contextual constraints and opportunities. The program so far has implemented mentoring as part of in-service capacity building in provincial and district hospitals. In the future, we plan to expand to health centers to ensure all tiers of the health system are supported. While our focus has been on in-service training, mentorship is potentially relevant and beneficial to pre-service education both for students and their educators, as has been demonstrated through training University of Health Sciences faculty in the capital Vientiane in the mentorship approach.

## Conclusion

Mentorship in Laos has transformed traditional models of education and learning. The collaborative, participatory peer coaching method differs from traditional hierarchical training models that have been shown to be ineffective (12). The clinically integrated and comprehensive approach in Laos ensures equal care for the mother and newborn at the time of birth. In essence, having mentors in every facility to support learning and skills development of mentees through low-dose, high-frequency coaching is effective and sustainable in the long term. Ensuring the approach is locally owned, developed by the mentors themselves, and is reflective and adaptive to context and constraints will ensure its longevity and sustainability in a competitive program climate.

The benefits of mentorship as a model of effective capacity building and supportive supervision relevant to both in-service and pre-service education are evident. Future research is needed to evaluate improvements in confidence and satisfaction of mentees and to understand how capacity building through mentorship translates into improved quality of care for mothers and newborns. The ultimate aim being to support the MoH reduce maternal and newborn mortality in Laos.

## Author Contributions

The author confirms to be the sole author and writer of this work and approves it for publication.

## Conflict of Interest Statement

The author declares that the research was conducted in the absence of any commercial or financial relationships that could be construed as a potential conflict of interest.

## Abbreviations

AMTSL, active management of the third stage of labor; EENC, early essential newborn care; MCH, maternal and child health; MCQ, multiple choice question; MCSP, Maternal Child Survival Program; MNH, maternal and newborn health providers; MoH, Ministry of Health; OSCE, objective structured clinical exam; PDR, People’s Democratic Republic; PHC, primary health care; RMNCH, Reproductive, Maternal, Newborn and Child Health; SCI, Save the Children International; SBA, skilled birth attendant; UNICEF, United Nations Child Fund; USAID, United States Agency for International Development; WHO, World Health Organization.
